# Cynaroside: a potential therapeutic agent targeting arachidonate 15-lipoxygenase to mitigate cerebral ischemia/reperfusion injury

**DOI:** 10.3389/fneur.2024.1490640

**Published:** 2025-02-14

**Authors:** Wenpeng Cao, Yufeng Hu, Xingyu Yu, Tingting Long, Baofei Sun, Shan Lei, Peng Xie, Wenfeng Yu

**Affiliations:** ^1^Department of Anatomy, School of Basic Medicine, Guizhou Medical University, Guiyang, China; ^2^Key Laboratory of Human Brain Bank for Functions and Diseases of Department of Education of Guizhou Province, Guizhou Medical University, Guiyang, China; ^3^Key Laboratory of Molecular Biology, School of Basic Medical, Guizhou Medical University, Guiyang, China; ^4^Class 5, Nursing, Grade 2023, Guizhou Medical University, Guiyang, China; ^5^Department of Physiology, School of Basic Medicine, Guizhou Medical University, Guiyang, China

**Keywords:** Cynaroside, cerebral ischemia/reperfusion injury, ferroptosis, inflammation, Alox15

## Abstract

**Introduction:**

Due to the anti-inflammatory and antioxidant properties of cynaroside (Cyn), it may be useful in the treatment of cerebral ischemia/reperfusion injury (I/R). This study aims to evaluate the effect of Cyn on cerebral ischemia/reperfusion injury.

**Methods:**

Transient middle cerebral artery occlusion model (tMCAO) and oxygen and glucose deprivation/reperfusion (OGD/R) microglia models were used to evaluate the effect of Cyn. The direct interaction between Cyn and Alox15 was investigated through bioinformatics, molecular docking and biolayer interferometry.

**Results:**

tMCAO mice treated with Cyn show improved neurological deficits, reduced infarct volume and edema, and inhibition of microglial activation. In addition, Cyn inhibited tMCAO-induced Alox15 expression. Cyn significantly reduced the overproduction of the M1 microglia-regulated pro-inflammatory cytokines NLRP3, ASC, and cleaved caspase-1, as well as the overproduction of IL-1β and IL-18, induced by tMCAO or OGD/R. Cyn also inhibits the expression of Tfrc, COX2, and Acsl4 in tMCAO and OGD/R-treated mice and BV-2 cells.

**Discussion:**

These results suggest that Cyn may attenuate cerebral ischemia/reperfusion injury by inhibiting Alox15 to reduce inflammation and reduce ferroptosis. This study reveals the underlying molecular mechanism of Cyn in the treatment of ischemic stroke.

## 1 Introduction

Cerebral ischemia/reperfusion (I/R) injury is a significant cause of morbidity and mortality worldwide (1). Current mainstream treatments, such as intravenous thrombolysis (IVT) and endovascular thrombectomy (EVT), have shown efficacy in restoring blood flow and improving clinical outcomes. However these treatments have limitations, including a narrow therapeutic window and potential for hemorrhagic complications (2). Additionally, the inflammatory response and oxidative stress associated with reperfusion can exacerbate neuronal damage (3). Therefore identifying a novel therapeutic approach has become a critical aspect of all research into cerebral ischemia/reperfusion injury.

When cerebral blood flow decreases, whether temporarily or permanently, it can cause cerebral ischemia, which can result in necrosis and cell death (3). The subsequent restoration of blood supply, known as reperfusion, may exacerbate the harm to ischemic brain tissue (4). Specifically, cerebral ischemia/reperfusion (I/R) injury is a progressive form of brain damage that is strongly associated with neurologic impairments, cognitive dysfunction, and significant injury to the brain (5). Additionally, reactive oxygen species (ROS) and oxidative stress are increased, as is neuroinflammation and neuronal apoptosis (6).

Astrocytes, another critical player in the central nervous system, have been shown to contribute significantly to the pathophysiology of cerebral I/R injury. These glial cells are “double-edged sword” in the pathological process of CIRI with both neurotoxic and neuroprotective effects on the central nervous system. These cells have been shown to contribute sighnificantly to mitigating oxidative stress (7). Compared to healthy brains, the presence of activated astrocytes is increased in ischemic brains, highlighting the potential involvement of activated astrocytes in the pathogenesis of cerebral ischemia/reperfusion injury (8).

Cynaroside is a flavonoid compound abundant in various plant species including honeysuckle, chrysanthemum, and celery, which exhibits a range of beneficial properties such as antioxidant and anti-inflammatory effects (9). Research has indicated that Cyn possesses antioxidative, anti-inflammatory, antibacterial, antiviral, anticancer, and other bioactive functions (10). Notably, recent studies have demonstrated the cardioprotective potential of Cyn *in vitro*, therefore Cyn also improved neurodegeneration diseases such as Alzheimer’s and polyglutamine disease by suppressing insulin/IGF-1 signaling pathway, yet there is a scarcity of literature regarding its neuroprotective effects (11, 12).

Cerebral ischemia–reperfusion (I/R) injury following thrombolysis in stroke results in the rapid demise of neurons in the ischemic penumbra (13). The pathophysiology of neuronal injury subsequent to cerebral ischemia is intricate, with a growing body of research indicating the significant involvement of the inflammatory response in this process (14). Elevated levels of inflammatory cytokines and pro-inflammatory mediators exacerbate neurological damage and facilitate ferroptosis in neural cells post-ischemia (15). Therefore, we infer that Cyn may serve as an intervention for ischemic stroke by preventing inflammation and ferroptosis.

Cynaroside has demonstrated strong antioxidant and anti-inflammatory effects, crucial in mitigating neuroinflammation and oxidative stress associated with CIRI. Its ability to regulate gene translation related to neuroprotection, reduce ROS, and inhibit pro-inflammatory cytokines underscores its potential as a therapeutic target for cerebral ischemia/reperfusion injury.

The Alox15 pathway, responsible for the metabolism of arachidonic acid, has been identified as a significant factor in cerebral ischemia injury (16). Studies have shown an upregulation of Alox15 expression in brain tissue following 24 h of ischemia, with knockdown of Alox15 resulting in reduced brain injury (17). These findings highlight the importance of investigating the molecular mechanisms underlying the role of Alox15 in cerebral ischemia injury. Further research is needed to elucidate the potential molecular mechanisms involved in iron dysregulation and inflammatory responses triggered by cerebral ischemia–reperfusion injury.

Using middle cerebral artery occlusion/reperfusion (MCAO/R) mouse models, and oxygen–glucose deprivation/reoxygenation (OGD/R)-induced BV-2 cells *in vitro*, this study aims to examine the effects and mechanisms of Cyn. In this study, the neuroprotective effects of Cyn against ischemic stroke, its modulation of ferroptosis and inflammation, as well as the regulatory pathways involved, will be investigated.

## 2 Results

### 2.1 Cyn protects against tMCAO-induced brain injury

**Figure 1:**
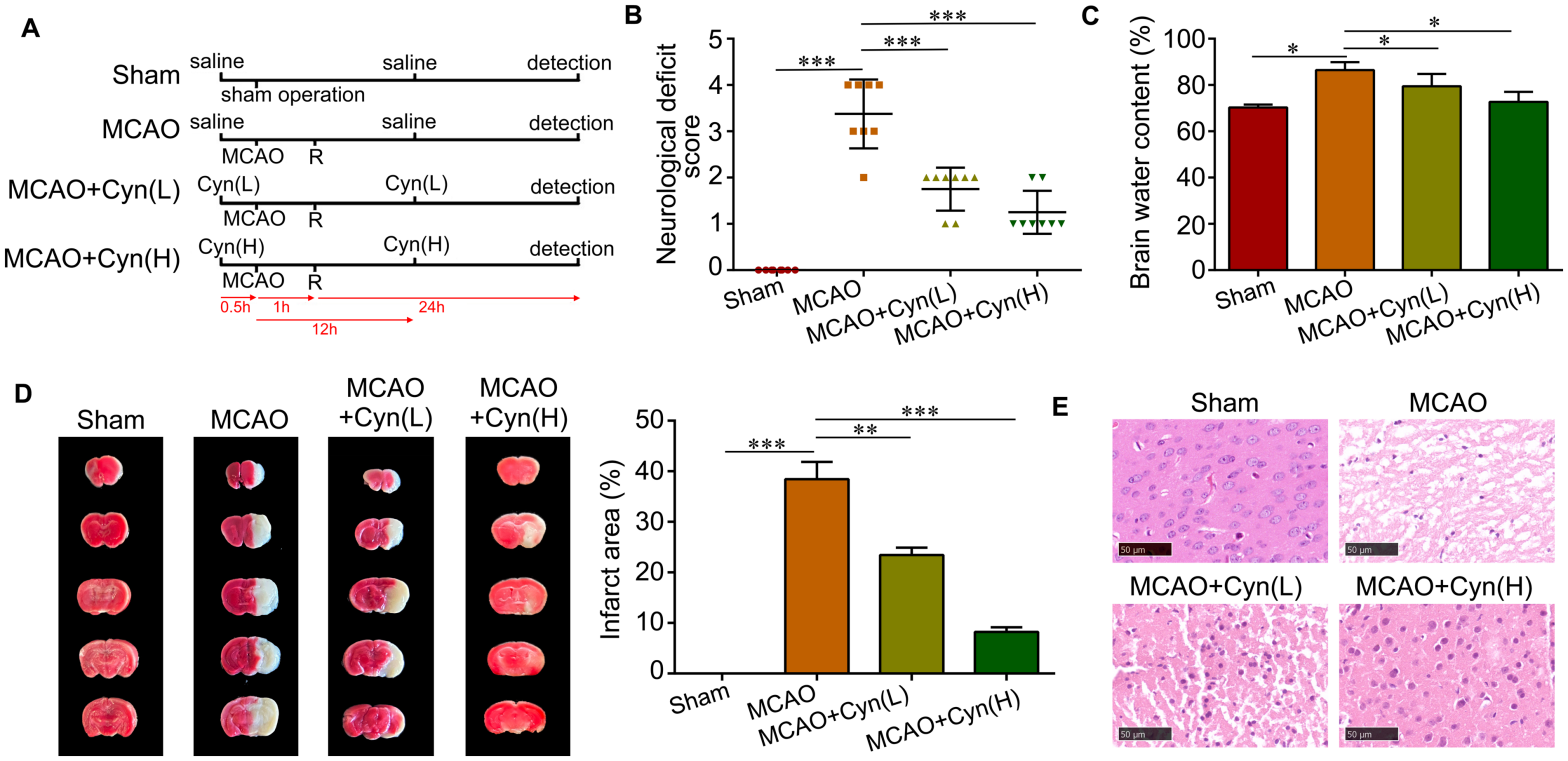
Cyn protects against tMCAO-induced brain injury and decreases microglia activation *in vivo*. **(A)** Diagram of animal model establishment and Cyn administration. A mouse model of tMCAO was established to mimic cerebral I/R injury *in vivo*. Mice were subjected to MCAO for 1 h followed by reperfusion (R) for 24 h. Cyn treatment at the dose of 10 (L) and 20 mg/kg (H) was performed 30 min before MCAO and 12 h after MCAO, respectively. After 24 h reperfusion, mice or brains were collected for further detection. **(B)** Assessment of neurological deficit scores. **(C)** Detection of brain water content. **(D)** Evaluation of infarct area with TTC staining. **(E)** Determination of neuron deficit with HE staining in the cerebral cortex region in the ipsilateral side of tMCAO. * represents *p* < 0.05; ** represents *p* < 0.01; *** represents *p* < 0.001. *n* = 3. The control group was used for comparison. Data are shown as mean ± SD.

Figure 1A illustrates the effects of Cyn on a mouse model of transient middle cerebral artery occlusion (tMCAO), which replicates cerebral I/R injury. The mice were assessed for their neurologic behavior based on their deficit scores after MCAO induction. A significantly higher deficit score was observed in MCAO mice compared to sham-operated control mice. Neurological deficit scores decreased significantly after treatment with Cyn (Figure 1B). Additionally, brain water content, which was elevated in MCAO mice, decreased significantly after Cyn treatment (Figure 1C). TTC staining was used to visualize the infarcted area, and the percentage of infarcted area in each brain section was calculated. Infarction on the ipsilateral side of the brain was not detected following the sham operation, whereas it was significantly increased following the MCAO. Treatment with Cyn inhibited tMCAO-induced infarction (Figure 1D). On the ipsilateral side of tMCAO, HE staining revealed neuronal loss in the cerebral cortex region. Cyn treatment mitigated this neuronal loss (Figure 1E).

### 2.2 Alox15 was identified as a key target of Cyn

**Figure 2:**
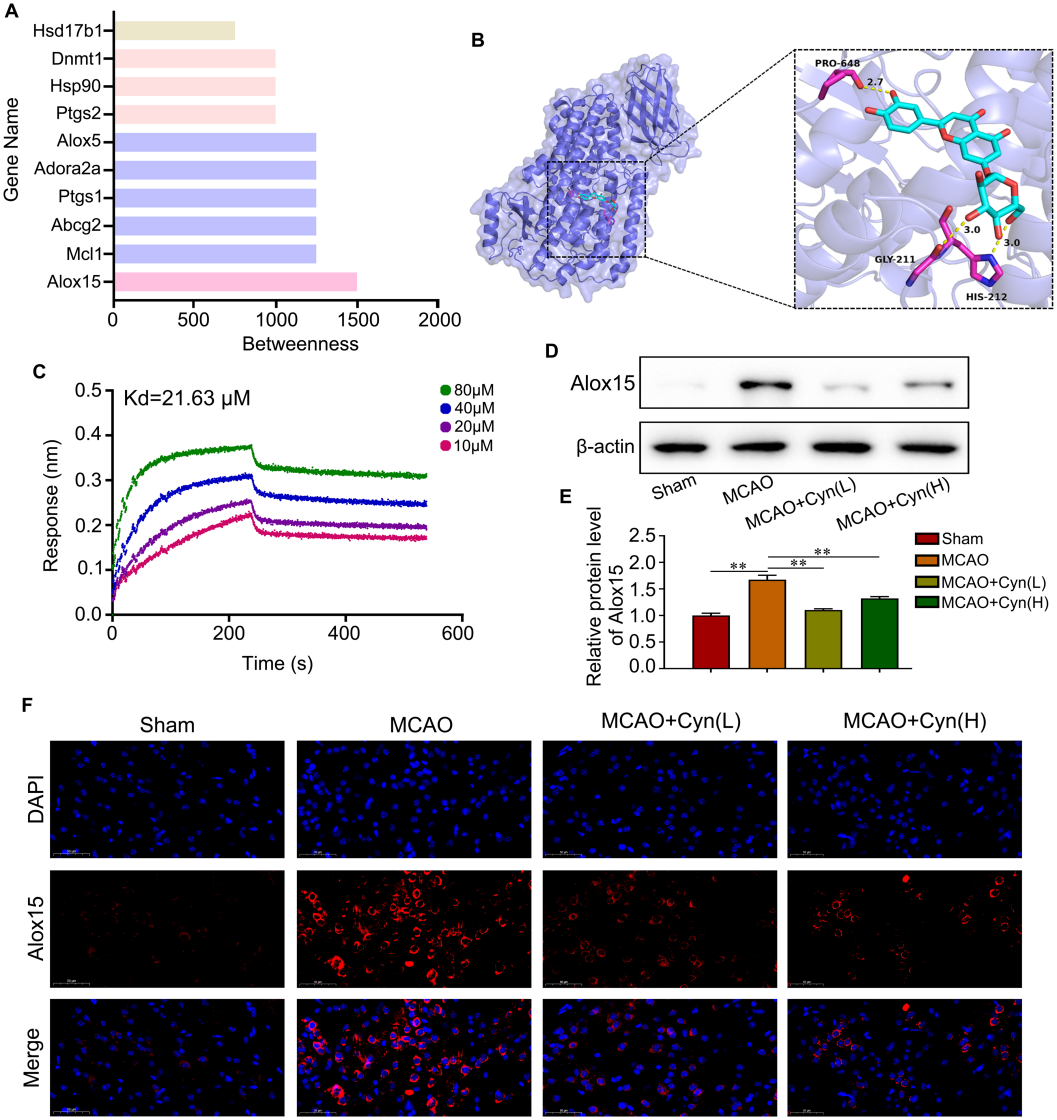
Alox15 was identified as a key target of Cyn. **(A)** The top 10 Cyn targets were visualized. **(B)** 3D-molecular docking of Cyn with OTUD7B. **(C)** Interaction of the Cyn with Alox15 by biolayer interferometry. **(D)** The protein expressions of Alox15 in cortex of cerebral ischemia reperfusion model with Cyn treatment. **(E)** Statistical analysis of the protein expression intensity of Alox15. **(F)** Immunofluorescence for Alox15 and counterstaining with DAPI in the cortex regions in the ipsilateral side of tMCAO. ** represents *p* < 0.01. *n* = 3. The control group was used for comparison. Data are shown as mean ± SD.

Using SwissTargetPrediction, we identified Alox15 as having the highest betweenness centrality (Figure 2A). Subsequently, we conducted an analysis of the binding model between Cyn and the Alox15 protein using molecular docking technology. The result of 3D-molecular docking illustrated that Cyn can effectively bind to the residues Gly211 and His212 of the Alox15 protein, demonstrating a stabilizing binding conformation (Figure 2B). Bio-layer interferometry (BLI) results demonstrated a kinetic association constant (Kd) of 21.63 μM for Cyn binding to Alox15 (Figure 2C). Western blotting and immunofluorescence analyses indicated an upregulation of Alox15 expression following tMCAO, which was subsequently downregulated in the presence of Cyn, with a more pronounced downregulation observed at higher doses of Cyn compared to lower doses (Figures 2D–F).

### 2.3 Cyn inhibited inflammation after tMCAO *in vivo*

**Figure 3:**
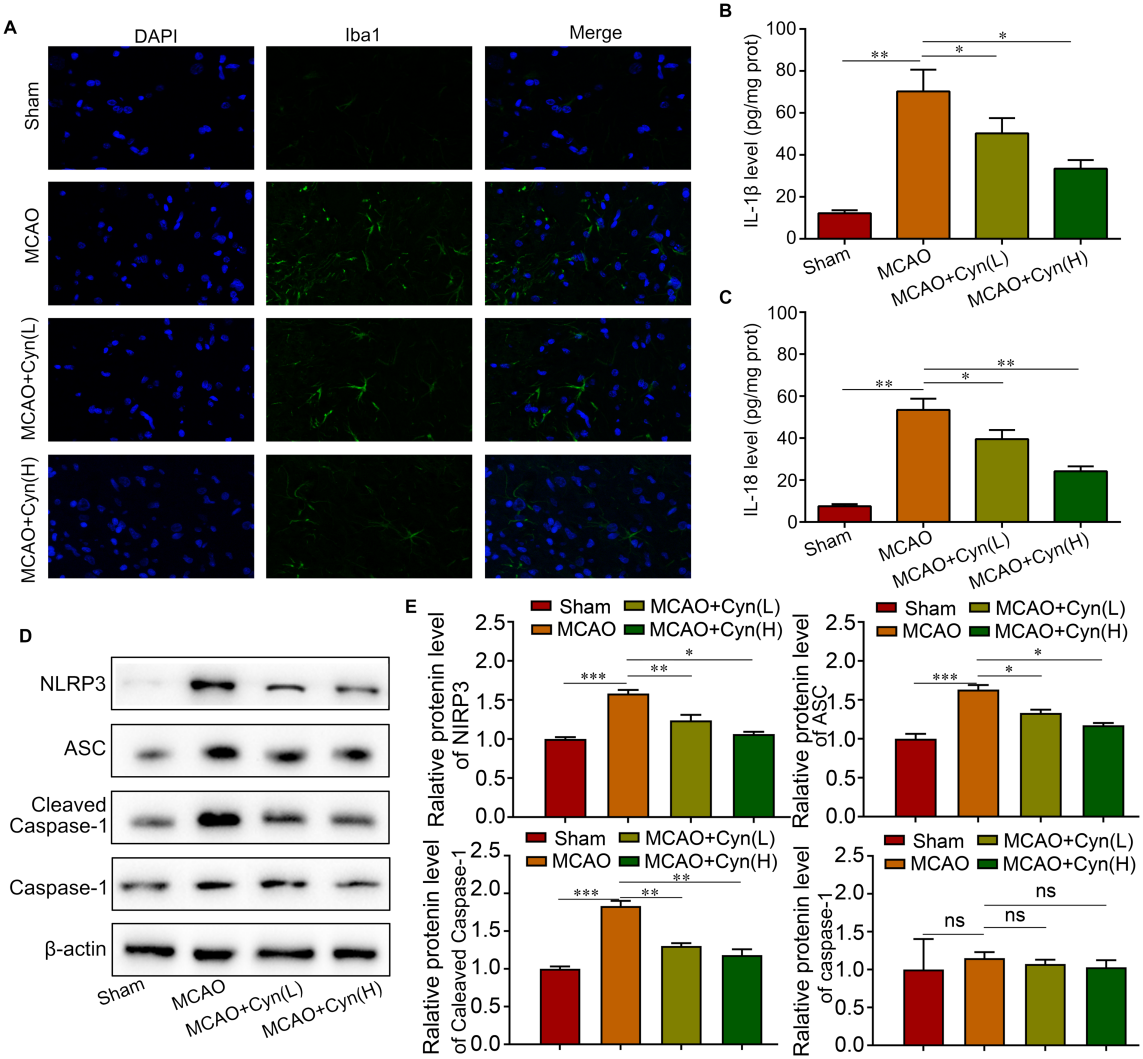
Cyn decreases tMCAO-induced NLRP3-inflammasome level *in vivo*. **(A)** Immunofluorescence for Iba1 and counterstaining with DAPI in the cortex regions in the ipsilateral side of tMCAO. **(B)** ELISA blot detected the secretion of IL-1β. **(C)** ELISA blot detected the secretion of IL-18. **(D,E)** Western blot detected the expression of NLRP3, ASC and caspasaae-1 and cleaved caspase-1. * represents *p* < 0.05; ** represents *p* < 0.01; *** represents *p* < 0.001. *n* = 3. The control group was used for comparison. Data are shown as mean ± SD.

Iba1 immunofluorescence staining showed that increased Iba1-positive microglia within the infarct region on the ipsilateral side of tMCAO, which decreased when treated with Cyn (Figure 3A). Cyn treatment in tMCAO mice decreased NLRP3-mediated inflammatory cytokine IL-1β and IL-18 release, as evidenced by ELISA tests, in the mice (Figures 3B,C). The levels of NLRP3, ASC, and cleaved caspase-1 were elevated following tMCAO, but decreased following Cyn treatment, However, full-length caspase-1 expression was not differentiated among the experimental groups (Figures 3D,E). These findings underscore the potential of Cyn in attenuating NLRP3 inflammasome-mediated inflammation subsequent to tMCAO.

### 2.4 Cyn suppressed tMCAO-induced ferroptosis *in vivo*

**Figure 4:**
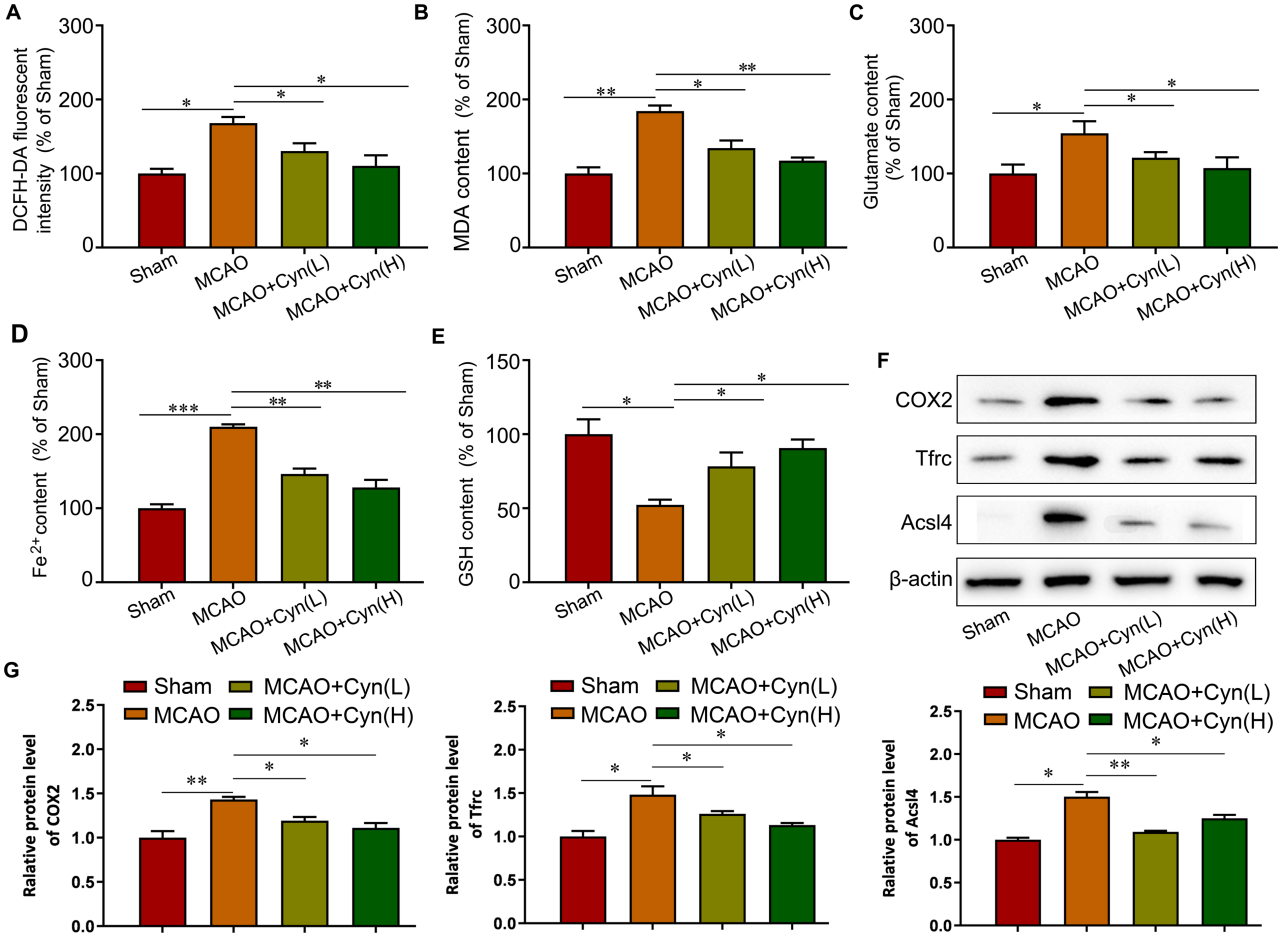
Cyn inhibited ferroptosis in MCAO/R mice. **(A)** Measurement and statistical analysis of ROS level. **(B)** Measurement and statistical analysis of MDA level. **(C)** Measurement and statistical analysis of glutamate level. **(D)** Measurement and statistical analysis of Fe^2+^ in brain tissue. **(E)** Measurement and statistical analysis of GSH level. **(F)** Western blot and quantification of Tfrc, Acsl4 and COX2 of mice. **(G)** Statistical analysis of the expression intensity of Tfrc, Acsl4 and COX2. * represents *p* < 0.05; ** represents *p* < 0.01; *** represents *p* < 0.001. *n* = 3. The control group was used for comparison. Data are shown as mean ± SD.

She et al. reported that factors involved in ferroptosis regulation include lipid peroxidation, Fe^2+^ levels and GPX4 activity (18). Rats subjected to MCAO/R for an hour, reactive oxygen species, malondialdehyde, glutamate, and iron levels significantly increased, while glutathione levels decreased. After treatment with Cyn, these changes were reversed (Figures 4A–E). Moreover, The Western Blot analysis revealed a significant upregulation in the expression levels of COX2, Tfrc, and Acsl4 following tMCAO, with a subsequent decrease in protein expression levels after treatment with Cyn (Figures 4F,G).

### 2.5 Cyn inhibited inflammation in OGD/R induced BV-2 cells

**Figure 5:**
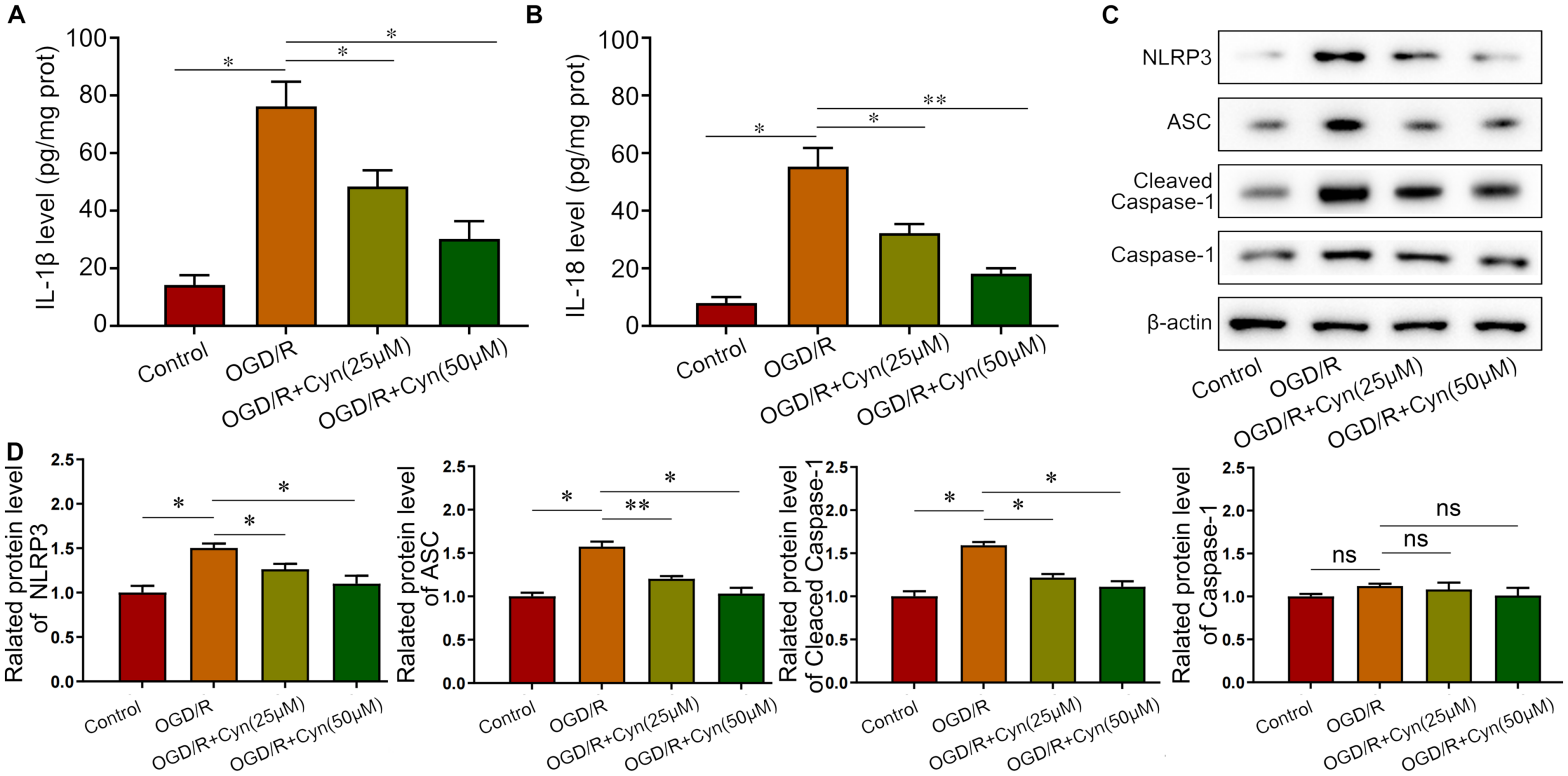
Cyn decreases OGD/R -induced NLRP3-inflammasome level *in vivo*. **(A)** ELISA detected the secretion of IL-1β after the treatment of Cyn. **(B)** ELISA detected the secretion of IL-18 after the treatment of Cyn. **(C)** Western blot detected the expression of NLRP3, ASC and caspase-1 and cleaved caspase-1 after the treatment of Cyn. **(D)** Statistical analysis of the expression intensity of NLRP3, ASC and caspase-1 and cleaved caspase-1. * represents *p* < 0.05;** represents *p* < 0.01. *n* = 5. The control group was used for comparison. Data are shown as mean ± SD.

We further investigated Cyn’s effects *in vitro* using BV-2 microglia cells with oxygen and glucose deprivation/reoxygenation (OGD/R) and varying concentrations of Cyn (25, 50 μM). ELISA analysis indicated an increase in the release of IL-1β and IL-18 mediated by NLRP3 inflammasome after OGD/R, which was attenuated by Cyn treatment (Figures 5A,B). Western blot analysis revealed that the expression levels of NLRP3, ASC, and caspase-1, key NLRP3 inflammasome proteins, were higher in OGD/R-treated cells but decreased in Cyn-treated cells. Full-length caspase-1 expression showed no significant difference (Figures 5C,D).

### 2.6 Cyn suppressed OGD/R-induced ferroptosis in BV-2 cells

**Figure 6:**
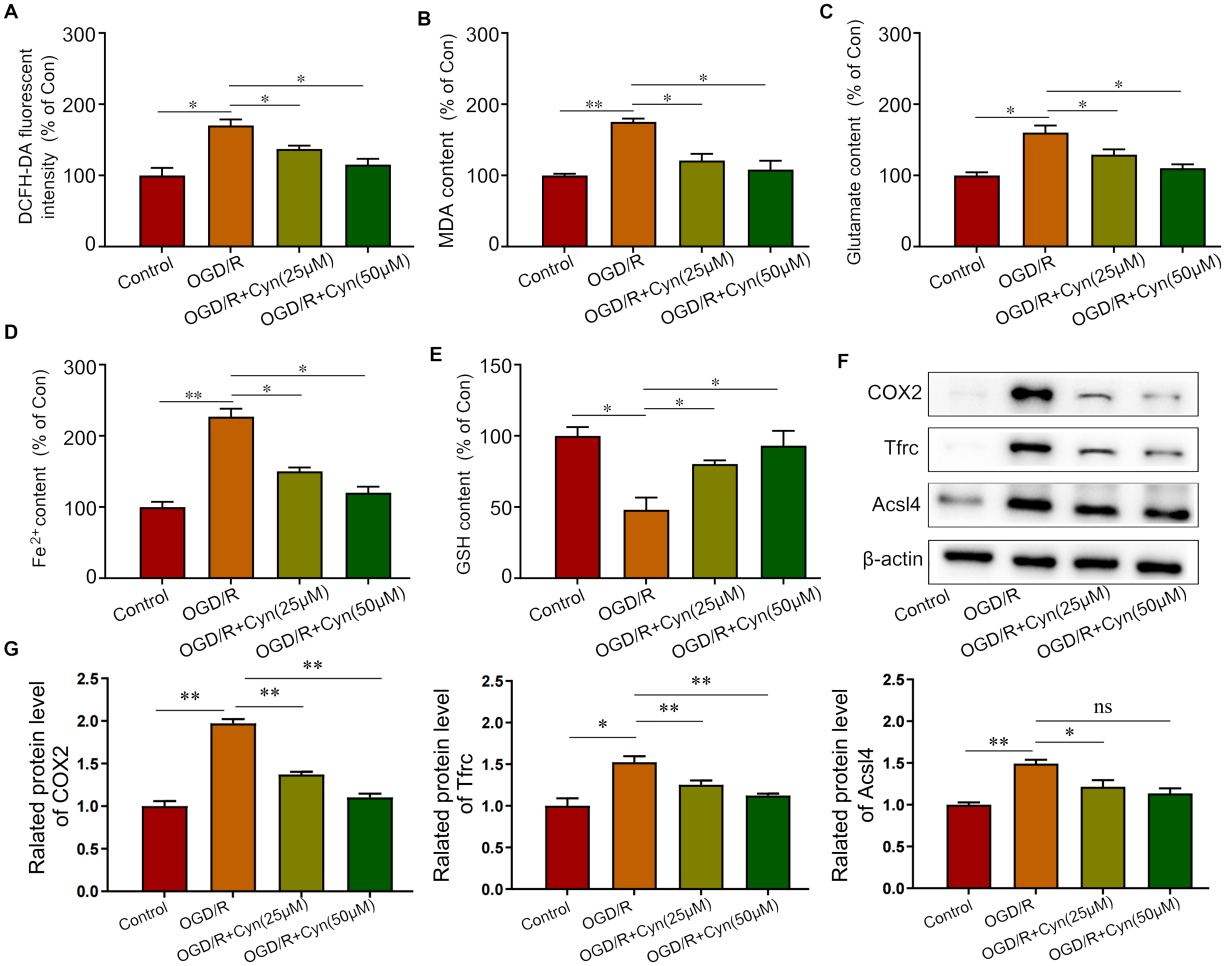
Cyn inhibited ferroptosis in OGD/R-treated BV-2 cells. **(A)** Measurement and statistical analysis of ROS level. **(B)** Measurement and statistical analysis of MDA level. **(C)** Measurement and statistical analysis of glutamate level. **(D)** Measurement and statistical analysis of Fe^2+^ in brain tissue. **(E)** Measurement and statistical analysis of GSH level. **(F)** Western blot detected the expression of Tfrc, Acsl4 and COX2 after the treatment of Cyn. **(G)** Statistical analysis of the expression intensity of Tfrc, Acsl4 and COX2. * represents *p* < 0.05; ** represents *p* < 0.01. *n* = 3. The control group was used for comparison. Data are shown as mean ± SD.

We observed that Cyn modulates ferroptosis post-OGD/R treatment by increasing ROS, MDA, glutamate, and iron levels, while reducing GSH levels, as evidenced by increased levels of ROS, MDA, glutamate, and iron. Remarkably, Cyn intervention reversed these alterations (Figures 6A–E). Correspondingly, Cyn downregulated COX2, Tfrc, and Acsl4 levels in OGD/R-exposed cells, consistent with our observations in mice (Figures 6F,G).

## 3 Discussion

Cerebral ischemia continues to be the primary contributor to morbidity and mortality on a global scale, with no pharmaceutical interventions demonstrating efficacy in clinical trials for ischemic stroke (19, 20). Consequently, the quest for viable and safe neuroprotective agents is of paramount importance and urgency. In recent times, natural herbal extracts have garnered significant interest due to their remarkable biological properties in various ailments (21). Cyn, a flavonoid compound prevalent in plants like honeysuckle, chrysanthemum, and celery, exhibits antioxidant, anti-inflammatory, and other beneficial effects (22, 23). Recent research has indicated that Cyn demonstrates cardioprotective effects *in vitro*, yet there is limited investigation into its neuroprotective properties (24, 25). These results align with previous studies, which also reported the anti-inflammatory properties of Cyn (23, 26). However, our findings differ from other studies, which did not observe significant improvements in neurobehavioral outcomes with Cyn treatment. This study presents novel findings demonstrating the neuroprotective effects of Cyn in rats with cerebral ischemia/reperfusion (I/R) injury. Experimental results illustrate that Cyn administration enhances neurological function and reduces brain edema following cerebral I/R injury. Additionally, histological analyses using TTC and HE staining further support the therapeutic efficacy of Cyn in rats subjected to middle cerebral artery occlusion (MCAO).

Mechanistically, Cyn enhances neurobehavior by modulating various signaling pathways. Specifically, Cyn has been shown to reduce neuroinflammation and oxidative stress (23, 27), which are critical factors in neurobehavioral deficits. Cyn plays a significant role in clinical impacts and gene translation regulation, contributing to its overall neuroprotective effects (28). Additionally, Cyn has been demonstrated to regulate gene translation by influencing the expression of genes involved in neuroprotection and inflammation.Our study provided additional insights into these mechanisms, revealing that Cyn’s neuroprotective effects are mediated through the inhibition of pro-inflammatory cytokine production and remitting total reactive oxygen species.

In recent years, the burgeoning discipline of network pharmacology has made a notable impact on the practice of traditional Chinese medicine (29, 30). A key aspect of network pharmacology involves the prediction of drug molecular mechanisms through intricate biological network models that elucidate interactions between targets and diseases (31). In this study, we utilized SwissTargetPrediction to forecast potential targets of Cyn, followed by the application of molecular docking technology to assess the binding affinity between Cyn and Alox15 targets. The study revealed that Cyn exhibited the highest binding affinity to Alox15, a finding corroborated by BLI experiments.

Arachidonate 15-lipoxygenase (Alox15) is an enzyme responsible for oxygenating polyunsaturated fatty acids and biomembranes, leading to the generation of multiple lipid metabolites in the Alox15 pathway (32). These small molecules act as potent signaling mediators implicated in various inflammatory diseases and cancers (33). Using animal and cell models, Lei demonstrated that Alox15 is involved in ferroptosis and inflammation after cerebral ischemia–reperfusion injury (17). Alox15 inhibitor therapy may protect neurons from SSAT1-induced iron death and loss of viability caused by TBH (tert-Butyl hydroperoxide) treatment, suggesting a role for Alox15 in brain injury pathogenesis (16). During cerebral ischemia–reperfusion injury, Alox15 may play a crucial role in regulating ferroptosis and inflammation. The present study also observed an increase in Iba1-positive microglia in the cortical regions of the ipsilateral brain hemisphere following tMCAO. In our study, tMCAO and oxygen–glucose deprivation/reoxygenation (OGD/R) increased NLRP3 inflammasome activation, resulting in elevated levels of pro-inflammatory cytokines, particularly IL-1β and IL-18 in M1 microglia. NLRP3 inflammasome components, such as NLRP3, ASC, and cleaved caspase-1, were reduced by Cyn treatment and inflammation-induced neuronal wound healing was decreased.

The present study also observed an increase in BV-2 microglia activity tMCAO and OGD/R, which linked to NLRP3 inflammasome activation. NLRP3 inflammasome components, such as NLRP3, ASC, and cleaved caspase-1, were reduced by Cyn treatment, leading to decreased levels of pro-inflammatory cytokines IL-1β and IL-18 in M1 microglia. This indicates that Cyn can modulate microglial activation and inflammation-induced neuronal damage.

Ischemia–reperfusion injury is associated with ferroptosis, a newly discovered cellular mechanism that contributes to neuronal death (34, 35). Apart from iron overload and lipid peroxidation, cerebral ischemia–reperfusion models have been shown to cause mitochondrial damage, including changes in mitochondrial membrane potential, cytochrome c release, and reactive oxygen species (ROS) generation (36, 37). Mitochondria play a crucial role in ATP synthesis through oxidative phosphorylation, leading to the generation of ROS, which is integral to the process of ferroptosis (38, 39). In the current investigation, it was observed that treatment with Cyn resulted in the inhibition of ALOX15 expression and a decrease in oxidative stress, accompanied by a reduction in reactive oxygen species (ROS) production, enhanced glutathione (GSH) activity, and decreased levels of malondialdehyde (MDA), iron, and glutamate in mice with transient middle cerebral artery occlusion (tMCAO) as well as BV-2 cells subjected to oxygen–glucose deprivation/reperfusion (OGD/R).

In conclusion, our results indicate that Cyn possesses neuroprotective properties against ischemic stroke by targeting Alox15, suppressing microglial activation and polarization, downregulating NLRP3-inflammasome components, and mitigating ferroptosis. Therefore, it is suggested that Cyn may represent a promising therapeutic strategy for cerebral I/R injury.

## 4 Materials and methods

### 4.1 Animal model of tMCAO

In this study, a total of 50 mice aged 10–12 weeks and weighing 30-35 g were obtained from Guizhou Medical University and housed under controlled conditions at 22°C and 45–55% humidity with a 12 h light/dark cycle and access to water and food *ad libitum*. Following a one-week acclimatization period, the mice were randomly assigned to one of four groups: (1) Sham, (2) MCAO, (3) MCAO + Cyn (L), (4) MCAO + Cyn (H). Consistent with prior studies (40, 41), embolization was performed on the right hemisphere of the brain using a 10 × microscope in a sterile environment. Induction and maintenance anesthesia were administered using 5% isoflurane and 2.5% halothane, respectively, prior to surgery (RWD Life Science Co, Shenzhen, China). The carotid sheath, common carotid artery, and internal carotid artery were exposed. Using a 0.26 mm nylon thread, a plug is inserted into the internal carotid artery through the common carotid artery and advanced into the intracranial space. After ligating and securing the plug, skin sutures are placed. Following a 150-min middle cerebral artery embolization procedure, the plug was retracted approximately 1 cm to restore blood flow. Mice in the sham group underwent identical surgical interventions without middle cerebral artery occlusion. Cyn (MCE, Wuhan, China) was administered intraperitoneally at doses of 10 mg/kg (low dose) or 20 mg/kg (high dose) 30 min before middle cerebral artery occlusion. For the MCAO group, saline was administered to mice. Animals displaying a 30% reduction in cerebral blood flow during MCAO, as well as those that expired during reperfusion, were excluded from subsequent experiments. An evaluation of neurological deficit scores was performed in mice after they had received 24 h of reperfusion. Afterward, mice were euthanized with 200 mg/kg pentobarbital, and brain tissues were collected.

### 4.2 Bio-layer interferometry

In the Octet RED96 System (ForteBio), the bio-layer interferometry (BLI) assay was used to determine Cyn’s binding affinity to Alox15. Biotinylation was performed according to the manufacturer’s instructions on ReadTM Streptavidin Biosensors to immobilize Alox15 proteins. The association of immobilized protein with flowing corosolic acid was detected with various concentrations of Cyn in the mobile phase. A running buffer is the PBS puffer with 0.1% DMSO and 0.02% Tween-20 was used for all experiments. Data were analyzed using ForteBio Data Analysis 9.0.

### 4.3 Evaluation of infarct volume with TTC staining

Following anesthesia, the brains were extracted and cryopreserved at −20°C for 15 min. Subsequently, the cerebellum and olfactory bulb were excised, and the brain tissue was sectioned into five equal parts in the coronal plane, each approximately 2 mm thick. The sections were then immersed in 0.4% TTC (Sangon Biotech Co., Ltd., Shanghai, China) solution at 37°C in a light-protected water bath, with regular flipping every 5 min to ensure uniform staining. Non-infarcted regions appeared red, while infarcted areas exhibited a gray-white hue. Following staining, the TTC solution was decanted, and the brain tissue was fixed in 4% paraformaldehyde. After a period of 6 h, the caudal side of each brain slice was examined using the Image-Pro Plus 6.0 system. The infarct size and total area of each slice were quantified, and the percentage of infarct area relative to the entire brain area was computed.

### 4.4 Detection of brain water content

After 24 h of reperfusion, the whole brain was harvested and weighed (wet weight). A second weight was obtained after drying the brain at 100°C. In order to determine the percentage of water in the brain, the formula is 100 × (wet weight - dry weight)/wet weight.

### 4.5 Protein evaluation

Protein expression levels were assessed using western blot analysis with antibodies against NLRP3 (Proteintech, No. 68102-1-Ig), ASC (Proteintech, No. 10500-1-AP), and COX2 (Proteintech, No. 66351-1-Ig), anti-caspase-1 (Proteintech, No. 22915-1-AP), anti- Alox15 (CST, No. 82129), Cleaved Caspase-1 (Bioss, No. bs-10743R), anti-Tfrc (Proteintech, No. 65236-1-Ig), anti-Acsl4 (Proteintech, No. 22401-1-AP) and anti-*β*-actin (Proteintech, No. 81115-1-RR).

### 4.6 Molecular docking

ChemDraw was used to generate Cyn’s structure, while Protein Data Bank (PDB) was used to retrieve proteins’ crystal structures. Subsequently, both the obtained protein structures and the Cyn structure were inputted into Autodock. Prior to docking, the protein structures underwent preparation steps involving the removal of water molecules and addition of hydrogen atoms. The docking process was executed using an algorithm, and the highest scoring binding model was selected for visualization of the interactions between Alox15 and Cyn.

### 4.7 Cells and cell treatment

After the BV-2 microglia cells were suspended in a sugar-free and serum-free medium, they were incubated in an hypoxic environment containing 0.2% O_2_ and 5% CO_2_ for 3 h before being switched to a sugar-containing medium containing 10% fetal bovine serum, and further incubated at 37°C with 5% CO_2_ for 24 h. Using standard culture conditions, control cells and cell models of cerebral ischemia–reperfusion injury were treated with various treatments according to experimental protocols.

### 4.8 Measurement of cellular ferroptosis levels

Brain tissues were removed, mechanically homogenized under an ice water bath, centrifuged at 2500 r/min for 10 min, and the supernatant was taken for determination. The Malondialdehyde (MDA), Reactive oxygen species (ROS), Glutathione peroxidase 4 (GPXs), Glutathione (GSH) and Fe^2+^ assay kit (Solarbio, Beijing, China) instructions were followed.

### 4.9 Enzyme-linked immunosorbent assay

Blood was collected from mice’s eye sockets overnight at 4°C then centrifuged at 3000 r/min for 5 min to separate the upper serum and lower serum. The ELISA kits were purchased from Absin (Shanghai) biotechnology company. The procedure that was followed was based on the instructions on the kit.

### 4.10 Western blot analysis

Rat brain tissue from the ischemia–reperfusion area or microglia proteins were obtained with the RIPA lysis buffer supplemented with protease and phosphatase inhibitors. The homogenates were then centrifuged at 12,000 rpm for 15 min at 4°C to remove debris. And then protein concentrations were determined using the BCA protein assay kit. Each sample of protein were isolated equally to 30 μg by electrophoresis and then transferred to PVDF membranes. The membranes were placed into primary antibodies against Alox15, NLRP3, ASC, Cleaved Caspase-1, Caspase-1, COX2, Trfc, Acsl4 and *β*-acting overnight at 4°C after being blocked with 5% non-fat dry milk in TBS-T at 36°C. Subsequently, the membranes were then washed with TBS-T and incubated with HRP-conjugated secondary antibodies for an hour at 36°C. Protein bands were visualized using an enhanced chemiluminescence (ECL) detection kit and quantified by densitometry using ImageJ software.

### 4.11 Neurological deficit assessment using longa score

Neurological function was assessed 24 h after reperfusion by an investigator blinded to the treatment groups. The scoring was based on a five-point scale, as described by longa et al. (42): 0, no neurological deficit (normal behavior); 1, failure to fully extend the contralateral forelimb; 2, circling to the contralateral side when held by the tail; 3, falling to the contralateral side; 4, no spontaneous walking with a depressed level of consciousness.

### 4.12 Statistical analysis

The data, with the exception of neurological deficit scores, are presented as mean ± standard deviation. Multivariate analysis was applied to assess the relationships between different variables, using techniques such as principal component analysis (PCA) to reduce dimensionality and identify the key patterns in the data ANOVAs and Tukey’s multiple comparison tests were used to assess the statistical significance of the differences between groups. GraphPad Prism 7.0 software was used to conduct all statistical analyses, and a significance level of 0.05 was set for all statistical analyses. Statistical analysis was conducted using Kruskal-Wallis and Dunn’s multiple comparison tests.

## Data Availability

The data used to support the findings of this study are available from the corresponding author upon reasonable request.
